# Formation of Surface Silver Nano-network Structures through Hot Electron Regulated Diffusion-limited Aggregation

**DOI:** 10.1038/s41598-019-43526-7

**Published:** 2019-05-06

**Authors:** Yu-Feng Yao, Shaobo Yang, Chin-Chou Teng, Keng-Ping Chou, Chi-Wu Liu, Yang Kuo, Yean-Woei Kiang, Chih-Chung Yang

**Affiliations:** 1 Institute of Photonics and Optoelectronics and Department of Electrical Engineering, National Taiwan University, No. 1, Section 4, Roosevelt Road, Taipei, 10617 Taiwan; 2 Department of Energy and Refrigerating Air-conditioning Engineering, Tung Nan University, 152 Beishen Road, Section 3, New Taipei City, 22202 Taiwan

**Keywords:** Nanoparticles, Nanoparticles

## Abstract

A surface Ag nano-network pattern is formed by first depositing Ag nanoparticles (NPs) on a conductive template, which has a certain defect structure, and then illuminating the Ag NPs with ultraviolet (UV) light in a moist environment. Such an Ag nano-network pattern consists of multiple connected Brownian trees (BTs), which are produced through the diffusion-limited aggregation (DLA) process. In the DLA process, diffuse Ag^+^ ions, which are generated by UV light illumination and dissolved by a thin adsorbed water layer on the surfaces of the Ag NPs and used GaN template, settle to form a BT through the combination with excited hot electrons migrating into the template from the Ag NPs. The lateral transport of hot electrons in the template is regulated by the distributions of threading dislocation and point defect cluster in the template, which eventually become the centers of BTs. The structure of a surface Ag nano-network can potentially serve as a transparent conductor.

## Introduction

The kinetics of the dimension changes of surface metal nanoparticles (NPs) has attracted much research attention. It has been reported that a sintering process can lead to the changes of metal NP dimension, including the merge of multiple NPs and the transfer of atoms from one NP to another. The sintering process includes two mechanisms: coalescence sintering and Ostwald ripening sintering^1–4^. In a coalescence sintering process, two NPs merge into a larger one when they contact each other or collide. In a process of Ostwald ripening sintering, individual atoms of one NP transport to another NP and vice versa. Eventually, the larger NP receives more atoms to become even bigger. Normally, a sintering process can occur only under certain extreme conditions, such as the irradiation of high-energy electrons or a high temperature (above several hundred °C).

The morphology change of a surface metal NP can also be observed under a milder condition through a mechanism related to the photochromism phenomenon^5^, in which the geometry of an Ag NP on a nano-porous TiO_2_ template is changed through the illumination of light at the wavelength of localized surface plasmon (LSP) resonance of the Ag NP. In a series of study on this subject, it was reported that under photo-excitation, intraband transition enhanced by LSP resonance could produce hot electrons. The hot electrons could overcome a potential barrier and migrate into the TiO_2_ template to leave Ag^+^ ions behind in an Ag NP. The Ag^+^ ions could be dissolved by the adsorbed water on the template and diffused in the water layer (1–2 nm in thickness) for a distance before recombining with electrons in the conduction band of TiO_2_ ^6,7^. This process led to the technique of reshaping an Ag NP through the excitations of different LSP resonance modes. At a hot spot of an LSP resonance mode, Ag atoms can be more easily ionized and hence more Ag^+^ ions are dissolved for changing the Ag NP geometry. The Ag portions to be removed are determined by the LSP resonance mode pattern and hence by the original metal NP geometry^8–14^. This technique of metal NP reshaping brought us with certain applications, including plasmonic manipulation of color^12^, photo-drawing of multi-color scattering image^14^, fabrication of porous Au NP^15^, and LSP-based sensing^16^. In this series of research, the study was focused on the geometry changes of individual metal NPs through LSP-resonance controlled reshaping.

Diffusion-limited aggregation (DLA) is a clustering process to form aggregations of diffuse particles^17^. In such a process, a particle undergoing random walk sticks onto an aggregate when it collides with a branch of the aggregate to eventually form a structure like plant vein or snowflake in the two-dimensional case. Such an aggregated structure is named as a Brownian tree (BT). DLA can be widely observed in natural phenomena and controlled chemical reactions, such as coral growth, lightning path, and electrodeposition. Models and numerical algorithms have been built for studying DLA behaviors^17–19^. Among various parameters controlling the formation of a BT in DLA, the mean free path or random walk step size of a diffuse particle is a key to determining the dimension of a BT^19^. A particle of a larger mean free path can more likely approach the center of a BT such that the BT dimension is smaller. Although DLA has been widely studied theoretically and numerically, experimental demonstrations with controlled parameters are limited, particularly in the two-dimensional case^20,21^.

In this paper, we demonstrate the formation of a surface Ag nano-network structure through a hot-electron regulated DLA process. Such a nano-network structure consists of multiple connected BTs to cover a large area. A BT is formed first by settling Ag^+^ ions near a defect cluster, either naturally formed or man-made, in the template due to hot electron capture by the defect cluster to form the center of the BT. The hot electrons originate from their migration over the potential barrier between Ag and template after their generation in surface Ag NPs through intraband transition under the illumination of ultraviolet (UV) light. The left behind Ag^+^ ions are dissolved by the adsorbed water on the surfaces of Ag NPs and template and then diffuse in the thin condensed water layer. Ag atoms settle at a location where diffuse Ag^+^ ions in the water layer recombine with transporting hot electrons in the template near its surface. The potential application of such a surface Ag nano-network structure is discussed. In this paper, although the similar behaviors of hot electron generation in Ag NPs and their migration into the conductive template, and the dissolution of Ag^+^ ions by the adsorbed water layer have been discussed in literature, the phenomena of Ag NP reorganization and nano-network formation through the DLA process, which is regulated by the defect structure in the template, have not been reported yet.

## Reorganization of Surface Silver Nanoparticles

**Figure 1:**
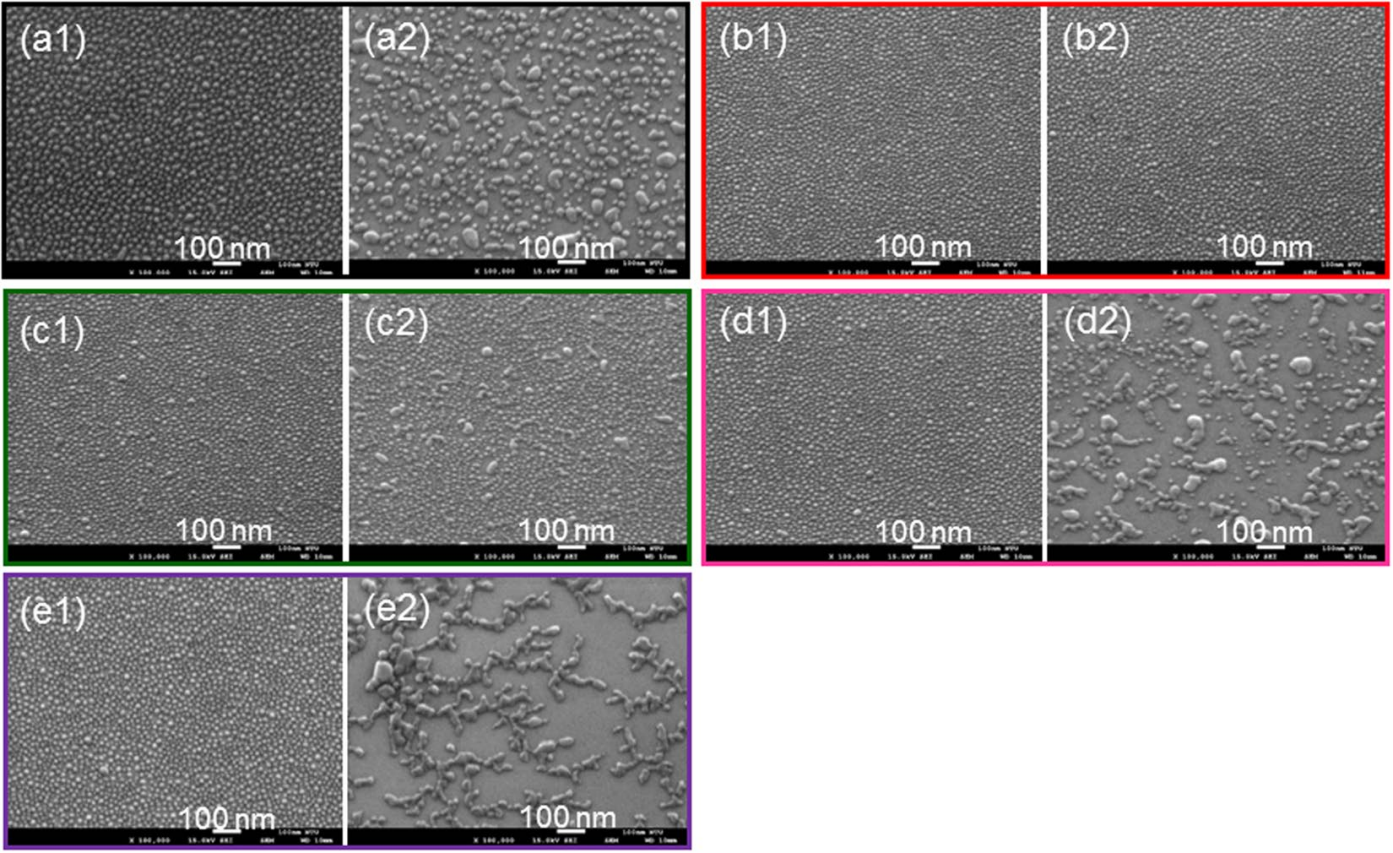
(**a1,a2**) SEM images of fresh surface Ag NPs and the Ag NP structure 16 hours later under the indoor natural condition, respectively, on a GaN template. (**b1,b2**) SEM images similar to parts (**a1,a2**), respectively, when the sample is illuminated by a red LED at 650 nm in wavelength. (**c1**,**c2**) SEM images similar to parts (**a1,a2**), respectively, when the sample is illuminated by a green LED at 520 nm in wavelength. (**d1,d2)**[(**e1,e2**)] SEM images similar to parts (**b1,b2**), respectively, when the sample is illuminated by an UV LED at 395 (367) nm in wavelength.

**Figure 2:**
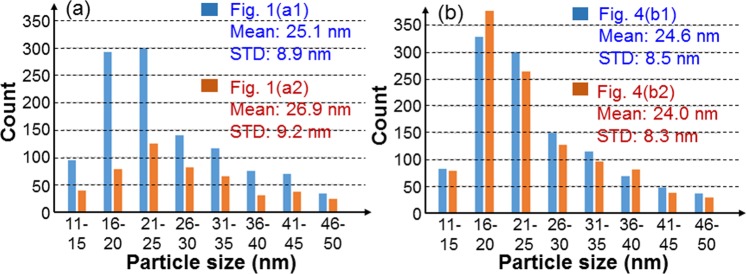
(**a**) Histograms of NP size distributions of the samples with the SEM images shown in Fig. 1(a1,a2). (**b**) Histograms of NP size distributions of the samples with the SEM images shown in Fig. 4(b1,b2). The mean and STD values of Ag NP size are also shown.

**Figure 3:**
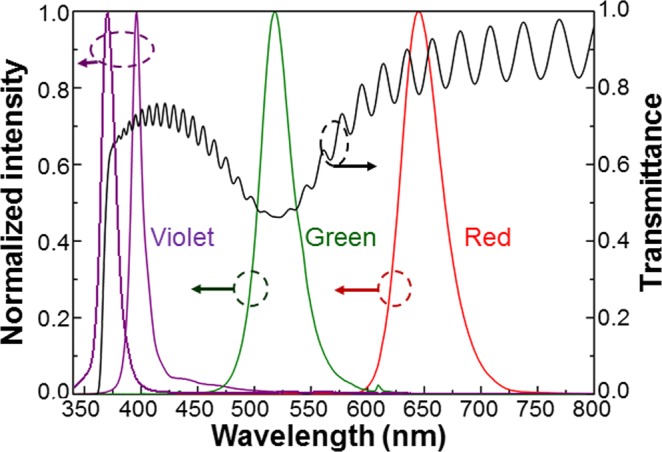
Transmission spectrum of a fresh Ag NP sample on a GaN template (with the right ordinate). With the left ordinate, the normalized spectra of the four LEDs used for Ag NP illumination are shown.

In Fig. 1(a1,a2), we show the scanning electron microscopy (SEM) images of fresh surface Ag NPs and the Ag NP structure 16 hours later under the indoor natural condition, respectively, on an un-doped Ga-face GaN template. The fresh Ag NPs are formed by depositing Ag of ~2.1 nm in thickness followed by a thermal annealing process at 180 °C for 30 min. Under the indoor natural condition, the sample is illuminated by sunlight coming through windows and light from fluorescent lamps at 25 °C. By comparing Fig. 1(a1,a2), one can see that the originally smaller Ag NPs aggregate to become larger NPs. For quantitatively confirming such an Ag NP aggregation phenomenon, we use the software of ImageJ (edition 1.8.0) to analyze the distribution of Ag NP size. Figure 2(a) shows the histograms of NP size distributions of the samples with their SEM images shown in Fig. 1(a1,a2). The mean and standard deviation (STD) values of Ag NP size are also illustrated here. By comparing the two data groups in Fig. 2(a), one can confirm that after the illumination of natural light for 16 hours, Ag NPs become larger. To specify the effective illumination spectral range for causing NP aggregation, we illuminate fresh Ag NP samples with light-emitting diodes (LEDs) of designated wavelengths in a dark chamber under the atmospheric condition. With the right ordinate, Fig. 3 shows the transmission spectrum of a fresh Ag NP sample on a GaN template. Here, the fast oscillation is caused by the Fabry-Perot effect of the sample. The transmission depression with the minimum around 520 nm in wavelength corresponds to the LSP resonance feature of Ag NPs on GaN. The zero transmission below ~365 nm is caused by GaN absorption. With the left ordinate, the four peaks in Fig. 3 show the normalized spectra of the four LEDs of four emission wavelengths used for Ag NP sample illumination. The emitted spectral peaks of the red, green, and two violet LEDs are located at 650, 520, 395, and 367 nm, respectively. The green light spectrum coincides with the LSP resonance feature. In Fig. 1(b1,b2), we show the SEM images of fresh Ag NPs and the Ag NP structure 16 hours later, respectively, when the sample is illuminated by the red LED with power density at ~2.65 mW/cm^2^. Here, one can see that the Ag NP morphology is unchanged after red light illumination. Figure 1(c1,c2) show the SEM images similar to those in Fig. 1(b1,b2), respectively, when the sample is illuminated by the green LED with about the same power density. Here, a weak change of Ag NP morphology is observed. Then, the morphology of fresh Ag NPs shown in Fig. 1(d1)[1(e1)] changes into what is shown in Fig. 1(d2)[1(e2)] after the illumination of the 395- (367-) nm LED for 16 hours with power density at 71.1 (6.1) mW/cm^2^. In these situations, the behaviors of Ag NP aggregation become significantly stronger. In particular, a certain Ag network structure is formed in Fig. 1(e2). Because natural light consists of a UV spectral component, one can conclude that UV illumination is a key factor for producing the behaviors of Ag NP aggregation or reorganization on GaN.

**Figure 4:**
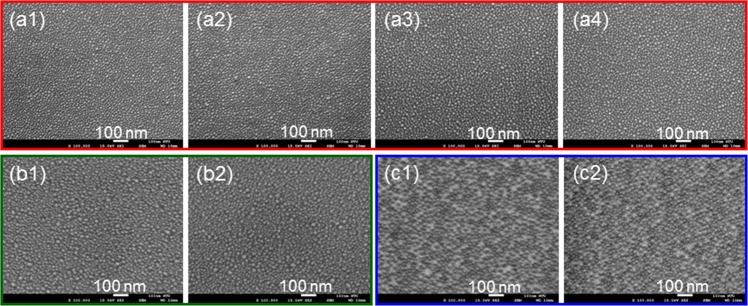
(**a1,a2**) SEM image of a fresh Ag NP sample on GaN and its image after 40-hour illumination of the 395-nm LED, respectively, when the sample is placed in a vacuum chamber of 10^−8^ torr in pressure. (**a3**,**a4**) Results similar to part (**a2**) when the sample is illuminated with ambient nitrogen and oxygen, respectively. (**b1,b2**) SEM image of a fresh Ag NP sample on SiO_2_ and its image after 40-hour illumination of the 395-nm LED in air, respectively. (**c1,c2**) SEM image of a fresh sample on sapphire and its image after 40-hour illumination of the 395-nm LED in air, respectively.

To understand whether there are still other crucial factors for producing the phenomenon of Ag NP reorganization, we illuminate fresh Ag NP samples with the 395-nm LED under different ambient vapor conditions and by using different templates. Figure 4(a1,a2) show the SEM image of a fresh Ag NP sample on GaN and its SEM image after 40-hour illumination of the 395-nm LED, respectively, when the sample is placed in a vacuum chamber of 10^−8^ torr in pressure. Figure 4(a3,a4) show the similar results when the sample is illuminated with ambient nitrogen and oxygen, respectively. Here, we can see that the Ag NP morphologies in Fig. 4(a1–a4) look almost the same, indicating that without ambient gas or with pure nitrogen or oxygen, Ag NP reorganization cannot be observed. Considering the composition of air, the only possible component for producing Ag NP reorganization is water vapor. Then, Fig. 4(b1,b2) show the SEM image of a fresh Ag NP sample on SiO_2_ and its image after 40-hour illumination of the 395-nm LED in air, respectively. Figure 2(b) shows the NP size histograms and their statistical data obtained from the SEM images in Fig. 4(b1,b2). From the comparisons of Ag NP morphology and NP size statistical result, one can see that on a SiO_2_ template, Ag NP reorganization cannot occur. Similar results are observed when we repeat the experiment with Ag NPs on a sapphire substrate, as shown in Fig. 4(c1,c2), in which the SEM image of a fresh sample and its image after 40-hour illumination of the 395-nm LED in air, respectively, are demonstrated. Therefore, we can conclude that besides the aforementioned UV illumination condition, two more critical conditions are required for producing the Ag NP reorganization phenomenon, including ambient water vapor and conductive template.

**Figure 5:**
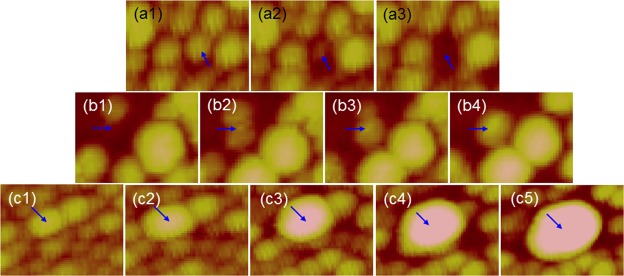
AFM images (100 nm × 75 nm) showing Ag NP reorganization behaviors of an Ag NP sample illuminated by the 395-nm LED in air. (**a1–a3**) AFM images of an area under illuminations for 0.5, 1, and 1.5 hours, respectively, to show the disappearance of an NP. (**b1–b4**) AFM images of another area under illuminations for 10, 11, 12, and 14 hours, respectively, to show the formation of a new NP. (**c1–c5**) AFM images of the other area under illuminations for 0.5, 1, 2, 3, and 4 hours, respectively, to show the merge of a few Ag NPs into a larger one.

For further understanding the Ag NP reorganization behavior, the atomic force microscopy (AFM) images of Ag NP structure during its evolution under UV LED illumination are continually taken. The three series of AFM image in Fig. 5 show the local Ag NP reorganization behaviors of an Ag NP sample on GaN illuminated by the 395-nm LED in air. In Fig. 5(a1–a3), we show the AFM images in an area of 100 nm × 75 nm in size under illuminations for 0.5, 1, and 1.5 hours, respectively. Here, the Ag NP pointed by the arrow in Fig. 5(a1) disappears completely in one hour. Figure 5(b1–b4) show the AFM images in another area of the same size under illuminations for 10, 11, 12, and 14 hours, respectively. As pointed by the arrows, a new Ag NP is formed in four hours. Figure 5(c1–c5) show the AFM images in the other area of the same size under illuminations for 0.5, 1, 2, 3, and 4 hours, respectively. Here, the pointed Ag NP merges the surrounding NPs to become a larger one.

**Figure 6:**
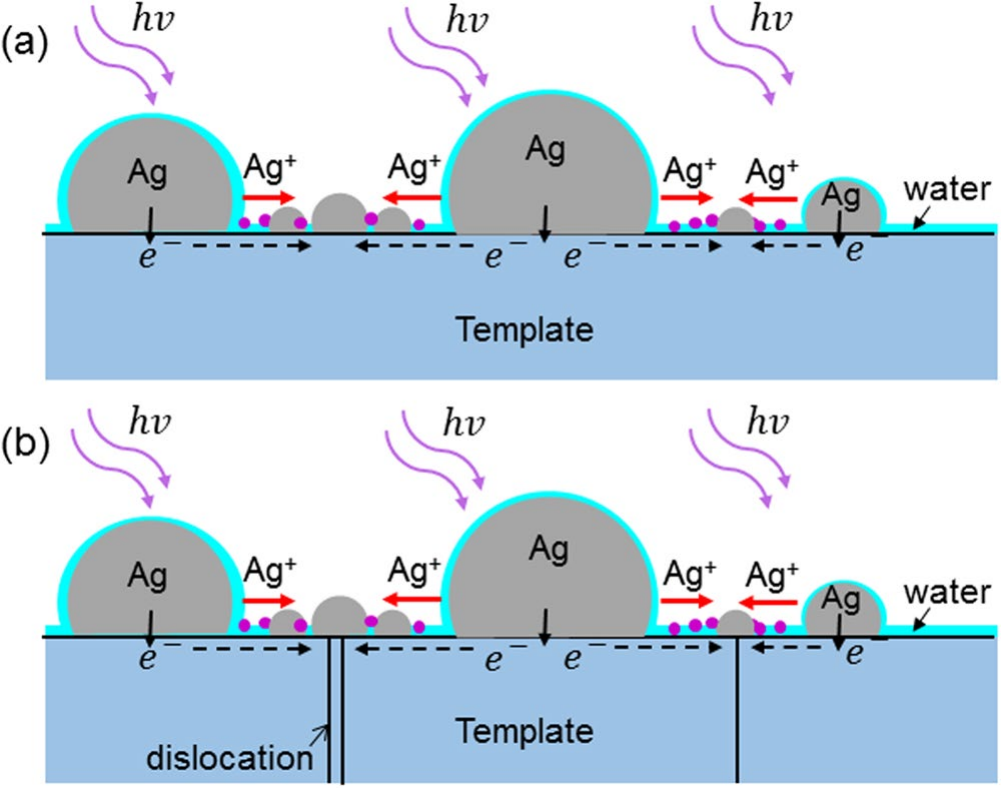
(**a**) Schematic demonstration of Ag NP reorganization mechanisms. (**b**) Schematic demonstration similar to part (**a**) except that threading dislocations are added for affecting electron transporting behavior.

**Figure 7:**
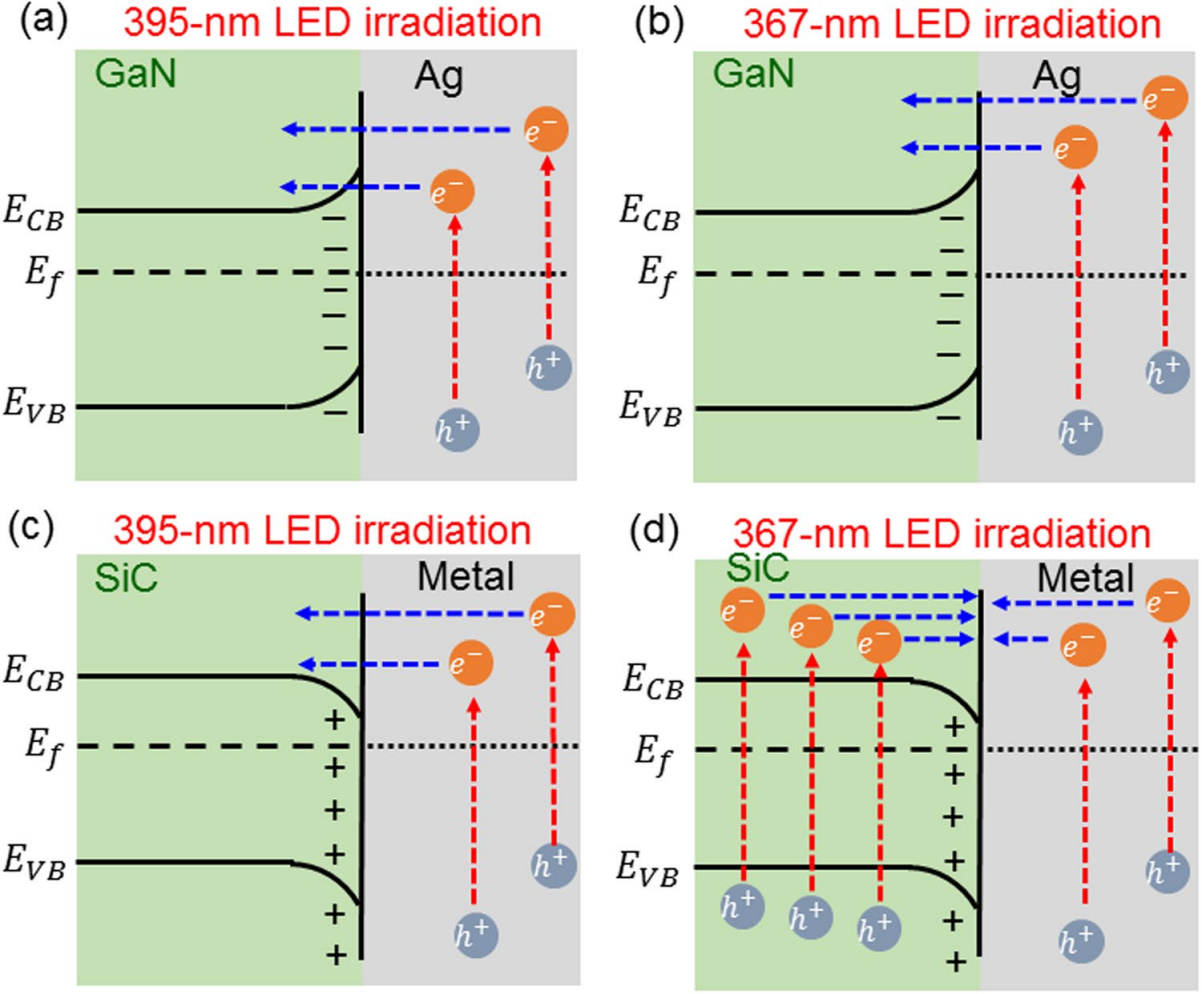
(**a–d**) Energy diagrams near the GaN/Ag and SiC/Ag interfaces when samples are illuminated by the 395- and 367-nm LEDs.

The mechanism of Ag NP reorganization is explained with the schematic illustration in Fig. 6(a). Here, the larger hemi-spherical NPs labeled by Ag represent the Ag NPs of a fresh sample. Under the illumination of UV light, hot electrons can be generated through intraband transition of Ag^22,23^. If the energy of those hot electrons is sufficiently high for overcoming the potential barrier between Ag and the template, they can migrate into the template for transporting there near its top surface, leaving behind Ag^+^ ions in an Ag NP^6^. Because of ambient moisture, the Ag NPs and template are covered by a thin layer (1–2 nm in thickness) of adsorbed water^7^. This thin water layer can dissolve Ag^+^ ions such that they can diffuse around on the template surface. The dissolution of Ag^+^ ions in an Ag NP by the water layer coarsens and reshapes the NP. When a hot electron in the conductive template near its surface meets an Ag^+^ ion on the template surface, they can combine to become an Ag atom and settle there. Through this process, effectively Ag atoms can move from one position to another on the template surface, leading to Ag NP reorganization. In this process, the moisture in air for producing the condensed water layer represents one of the key factors. Also, the choices of illumination wavelength and template material are important for Ag NP reorganization to occur. The high photon energy of illuminating UV light is needed such that hot electrons can have sufficient energy to overcome the potential barrier between Ag and template. Figure 7(a,b) show the energy diagrams near the GaN/Ag interface when Ag NPs are illuminated by the 395- and 367-nm LEDs, respectively. In these situations, hot electrons can overcome the potential barrier between GaN and Ag for entering GaN. In a previously reported study, Ag NPs were formed on a TiO_2_ template for demonstrating the photochromism phenomenon through the similar process of illuminating the Ag NPs with green light to induce LSP resonance on Ag NPs^5^. In their situation, the potential barrier between Ag and TiO_2_ is relatively lower such that the hot electrons excited by green light can overcome it for migrating into the TiO_2_ template. However, in our situation, although we do not exactly know the potential barrier level between GaN and Ag, it must be higher than 2.38 eV (520 nm in wavelength) and lower than 3.14 eV (395 nm in wavelength). Therefore, UV illumination can guarantee hot electron migration into GaN template for producing the Ag NP reorganization phenomenon. The lower potential barrier between TiO_2_ and Ag allows us to use visible light, whose spectrum is close to the LSP resonance feature of Ag NPs, for exciting hot electrons of high enough energy and hence generating Ag^+^ ions in Ag NPs. In this situation, the LSP resonance can enhance hot electron generation and hence the photochromism phenomenon^22,24–26^. Although the use of the 395- or 367-nm LED does not excite strong LSP resonance, the amount of excited hot electrons is sufficient for producing the observed Ag NP reorganization behaviour. It is noted that although the recombination of transporting hot electrons in the template and diffuse Ag^+^ ions in the condensed water layer was claimed to be the major mechanism for Ag NP reorganization, Ostwald ripening may also occur at the same time in the reorganization process.

**Figure 8:**
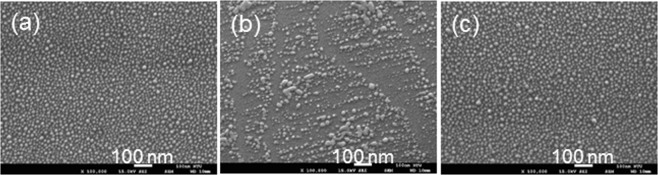
(**a–c**) SEM images of a fresh Ag NP sample on a Si-face 4H-SiC template, the Ag NP structures after the illuminations of 395- and 367-nm LEDs, respectively, for 16 hours in air.

The bandgap of the conductive template is also an important factor for producing the Ag NP reorganization behavior. Figure 8(a–c) show the SEM images of a fresh Ag NP sample on a Si-face 4H-SiC template, the Ag NP structures after the illuminations of 395- and 367-nm LEDs, respectively, for 16 hours in air. As shown in Fig. 8(b), by illuminating with the 395-nm LED, Ag NPs are reorganized although the mechanism causing the line structure is unclear. However, as shown in Fig. 8(c), by illuminating with the 367-nm LED, Ag NP reorganization does not occur. This result is quite different from what is shown in Fig. 1(e2) on a GaN template. The different results are caused by the different bandgaps between GaN and SiC. The bandgap of GaN is around 3.42 eV, corresponding to a wavelength (363 nm) slightly shorter than 367 nm such that the photon of neither UV LED can significantly excite electron in GaN. The bandgap of 4H-SiC is around 3.26 eV, corresponding to a wavelength (380 nm) longer than 367 nm but shorter than 395 nm. Therefore, electrons can (cannot) be generated in SiC when the 367- (395-) nm LED is used for illumination. Figure 7(c,d) show the energy diagrams near the SiC/Ag interface when Ag NPs on SiC are illuminated by 395- and 367-nm LEDs, respectively. Under both LED illumination conditions, excited hot electrons in Ag NPs can overcome the potential barrier between SiC and Ag for entering SiC. However, electrons generated in SiC can migrate into Ag NPs when the sample is illuminated by the 367-nm LED. In this situation, the two-way migration of electron can suppress the generation of Ag^+^ ions in Ag NPs such that few Ag^+^ ions can diffuse around in the water layer for producing Ag NP reorganization. For further study on Ag NP reorganization, GaN is a good choice of template for either UV LED emission wavelength.

## Formation of Silver Nano-Network Pattern

**Figure 9:**
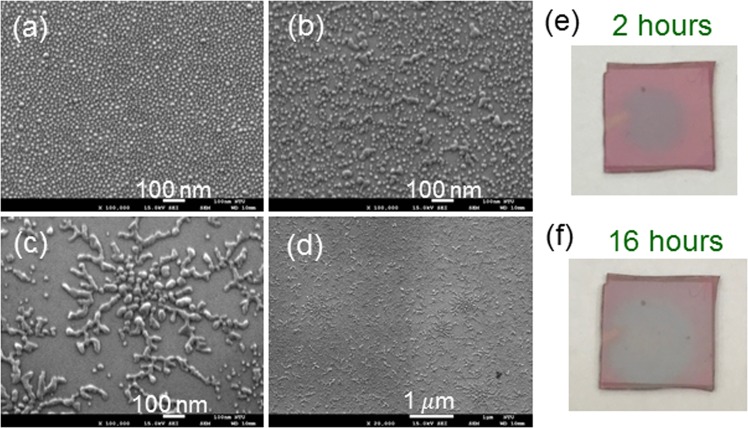
(**a–c**) SEM images of a fresh Ag NP sample on GaN, its Ag NP structures after the illuminations of the 395-nm LED for 2 and 16 hours, respectively, when ambient air humidity is ~40%. (**d**) SEM image in the case of 16-hour illumination with a smaller magnification. (**e,f**) Photographs of the samples with 2- and 16-hour illuminations, respectively.

**Figure 10:**
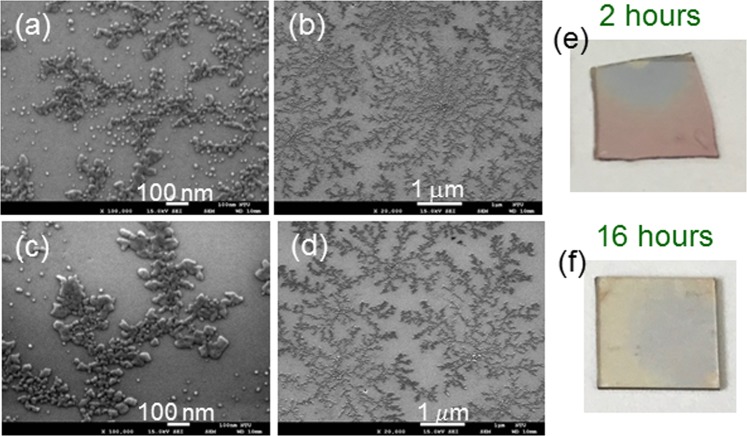
Results similar to Fig. 9 when ambient air humidity is increased to ~80%. (**a,b)[(c,d**)] SEM images of different magnifications under the condition of 2- (16-) hour illumination. (**e,f**) Photographs of the samples with 2- and 16-hour illuminations, respectively.

**Figure 11:**
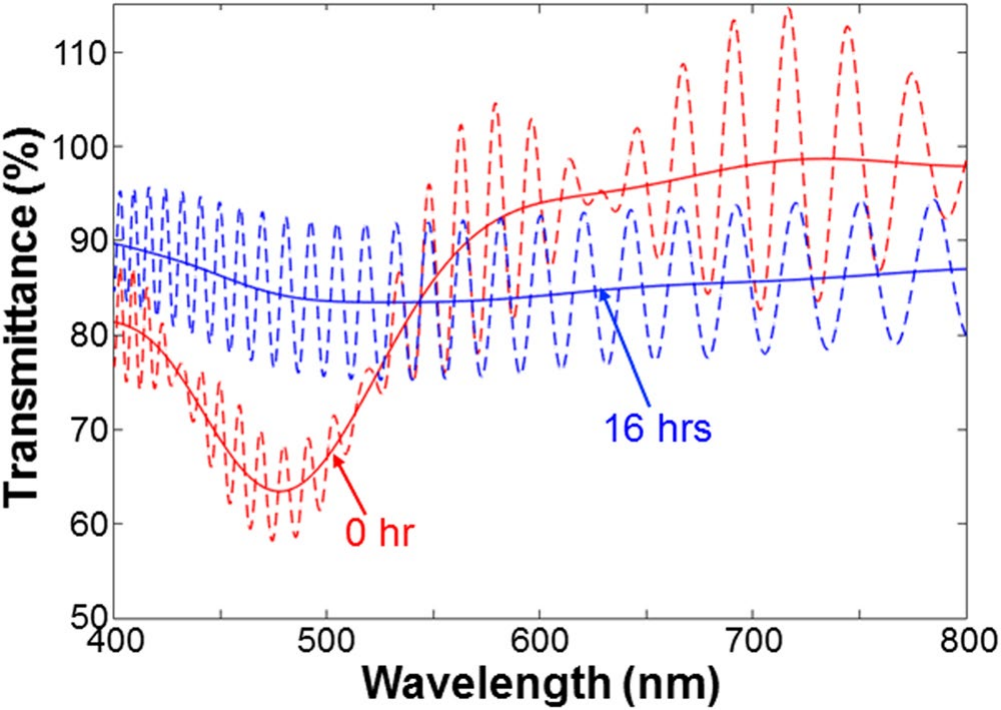
Transmission spectra of the Ag NP samples before (0 hr, red curves) and after 16-hour (16 hrs, blue curves) illumination shown in Figs 9(a) and 10(d), respectively. The oscillating dashed curve in each group represents the measured data with Fabry-Perot oscillation. The smooth curve is obtained by filtering the fast oscillation.

Under certain conditions, the reorganized Ag NPs form network patterns. Figure 9(a–c) show the SEM images of a fresh Ag NP sample on GaN, its Ag NP structures after the illuminations of the 395-nm LED for 2 and 16 hours, respectively, when the ambient air humidity is fixed at ~40%. Figure 9(d) shows the SEM image of a smaller magnification in the case of 16-hour illumination. As shown in Fig. 9(c), the Ag distribution shows a network pattern with a center of a higher Ag density and extended branches from the center. In Fig. 9(d), one can see multiple network patterns with a center in each pattern. Figure 9(e,f) show the photographs of the samples with 2- and 16-hour illuminations, respectively. Here, the color of the illuminated spot turns from pink into grey. Figure 10(a–f) show the similar results when the ambient air humidity is increased to ~80%. Here, Fig. 10(a,b) [10(c,d)] show the SEM images of different magnifications in the case of 2- (16-) hour illumination. One can clearly see the individual network patterns like BTs formed through the DLA process. Figure 10(e,f) show the photographs of the samples with 2- and 16-hour illuminations, respectively. After illumination for 16 hours, the sample becomes semi-transparent. The comparison between Figs 9(a–f) and 10(a–f) indicates that a higher ambient moisture, which can lead to a thicker adsorbed water layer on the sample surface, results in a higher Ag NP reorganization speed. Figure 11 shows the transmission spectra of the Ag NP samples before (labeled by “0 hr”, red curves) and after 16-hour (labeled by “16 hrs”, blue curves) illumination shown in Figs 9(a) and 10(d), respectively. Here, the oscillating dashed curve in each group represents the measured data with Fabry-Perot oscillations. The smooth curve is obtained by filtering the fast oscillation. Before LED illumination, the transmission depression with the minimum at 478 nm corresponds to the LSP resonance feature of fresh Ag NPs. This LSP resonance occurs at a shorter wavelength, when compared with that in Fig. 3, because of the relatively smaller Ag NP size. After 16-hour LED illumination, this depression disappears to show an essentially flat transmission spectrum of ~84% in transmittance minimum within the range between 400 and 800 nm.

**Figure 12:**
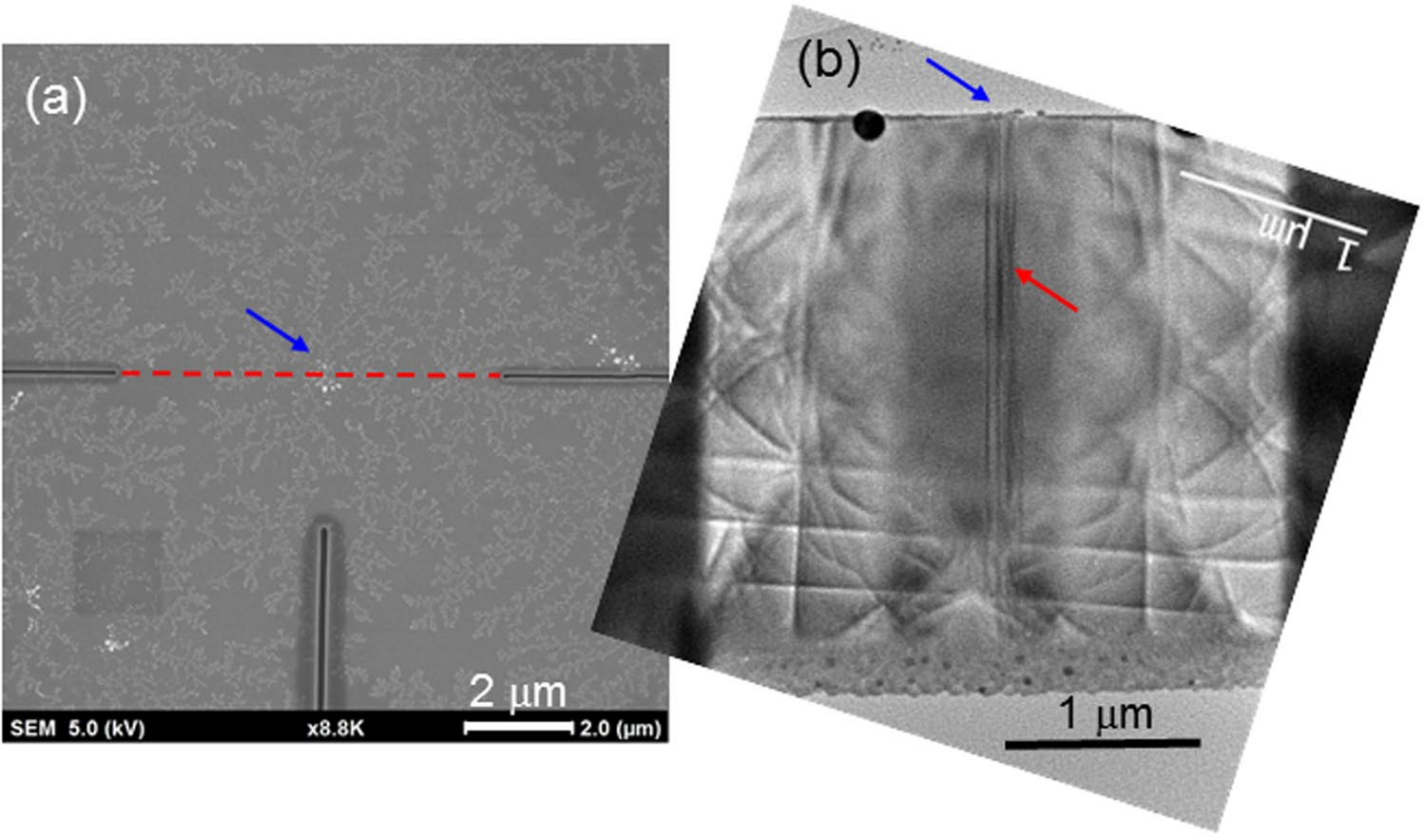
(**a**) SEM image of an Ag NP sample with multiple BTs under FIB process for preparing a TEM specimen. (**b**) Cross-sectional TEM image right below a BT center.

As shown in Fig. 10(b,d), a local network pattern is formed around a center through the DLA process. The question is what structure in a GaN template can serve as such a BT center. A transmission electron microscopy (TEM) observation of the GaN template around a BT center can help in answering this question. Figure 12(a) shows a plane-view SEM image of an Ag NP sample with multiple BTs under focused ion beam (FIB) process for preparing a TEM specimen. The two horizontal and one vertical dark line segments correspond to the scanning lines of FIB. We use the red dashed line to connect the two horizontal FIB scanning line segments for indicating that the FIB cutting passes the center of a BT, which is pointed by the blue arrow. In other words, using this specimen, we can obtain a cross-sectional TEM image right below the BT center, as demonstrated in Fig. 12(b). Here, right below the BT center, which is pointed again by the blue arrow at the top, there are a few dark lines. Each dark line corresponds to a threading dislocation in GaN. Because of the lattice mismatch between epitaxial GaN and sapphire substrate, threading dislocations are formed in GaN for releasing strain energy. Threading dislocations can propagate from bottom to top in a GaN layer. Unless a special growth technique is used, threading dislocations of 10^8^–10^9^ cm^−2^ in planar density are typically formed in growing GaN on sapphire substrate^27^. In the current study, a threading dislocation can trap hot electrons. Therefore, in GaN, free electrons tend to move toward a threading dislocation, including the hot electrons from Ag NPs, as schematically illustrated in Fig. 6(b). With a high-density electron distribution near a threading dislocation, Ag^+^ ions nearby are first neutralized and settled to form the high Ag density center. Then, with the tendency of electron transport toward the threading dislocation, Ag^+^ ions on electron transport paths are neutralized and settled to form the BT branches through the DLA process. Therefore, threading dislocations can serve as the centers of BTs. It is believed that a cluster of point defects can also have the similar function of serving as the center of a BT.

**Figure 13:**
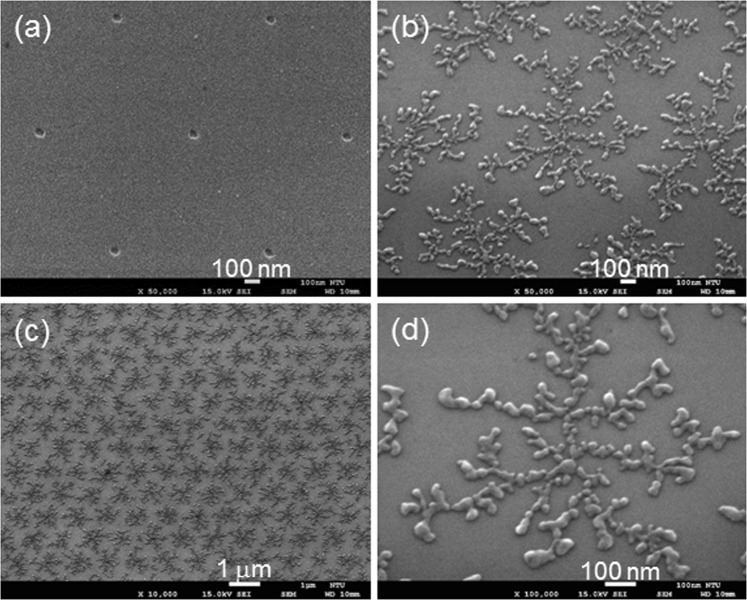
(**a**) SEM image of a hole array on a SiO_2_ mask. (**b**) SEM image showing the Ag NP structure after Ar^+^ ion bombardment, SiO_2_ liftoff, Ag deposition, and 16-hour illumination of the 395-nm LED. (**c**) SEM image of a larger area to demonstrate the patterned array of Ag BT. (**d**) Magnified SEM image of the central BT in part (**b**).

To confirm the aforementioned claim about BT center, we use electron-beam lithography and reactive ion etching to fabricate a triangularly patterned hole array of ~60 nm in hole diameter and 1 μm in pitch on a 20-nm SiO_2_ mask, which is deposited on a GaN template. Figure 13(a) shows the SEM image of the hole array on the SiO_2_ mask. This template is bombarded by Ar^+^ ions of 70 W in radio-frequency power for 30 sec in a reactive ion etching chamber to form point defects in GaN at the positions of patterned holes. Then, the SiO_2_ mask is removed through BOE (buffered oxide etch) dipping. Next, 1.6-nm thick Ag is deposited to form Ag NPs for the NP reorganization experiment under the condition of ~80% in ambient air humidity. After 16-hour illumination of the 395-nm LED, we obtain the Ag pattern shown in Fig. 13(b). Here, one can observe seven BTs with their centers corresponding to the seven patterned holes in Fig. 13(a). This correspondence confirms that a defect cluster, either naturally-formed or man-made, can serve as the center for forming a BT. Figure 13(c) shows the SEM image of a larger area to demonstrate the patterned array of Ag BT. Figure 13(d) shows the magnified SEM image of the central BT in Fig. 13(b). The Ag pattern shown in Fig. 13(c) can result in surface current conduction if individual BTs can be connected. Because of its high transparency, such a surface Ag nano-network structure can potentially be used as a transparent conductor.

**Figure 14:**
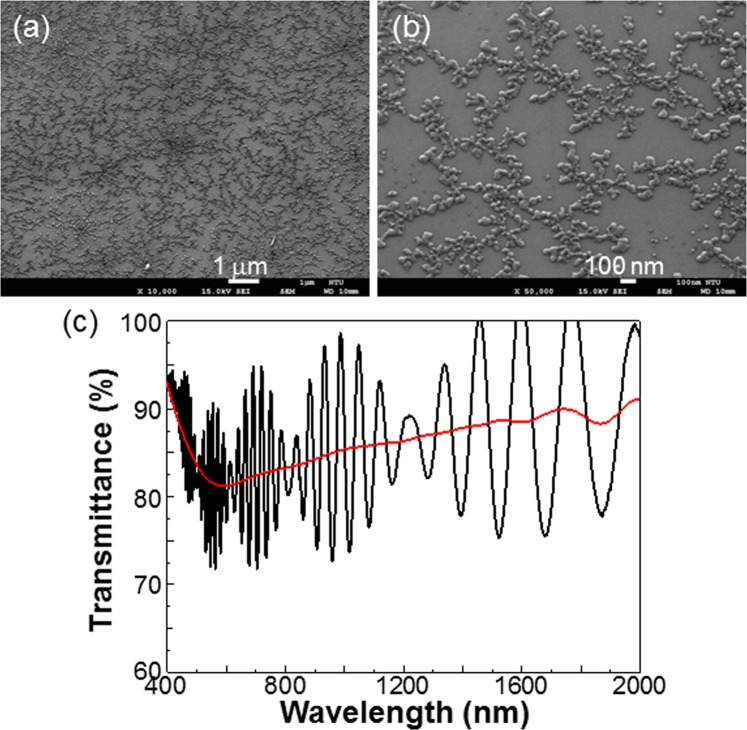
(**a**) SEM image of an Ag nano-network structure. (**b**) Magnified SEM image of the Ag nano-network structure. (**c**) Transmission spectra of this Ag network structure, including the oscillating measured data (black) and smooth fitted result (red).

Besides the formation of localized defect clusters for serving as the centers of BTs as shown in Fig. 13(a), the production of distributed point defects through large-area Ar^+^ ion bombardment with a lighter dosage or the generation of a thin oxide layer on the template surface can help in forming a well-connected Ag nano-network structure. The distributed point defects can serve as minor electron trap centers to form small Ag NP clusters for connecting major Ag BTs such that a large-area network structure can be fabricated. The thin oxide layer can be generated through a thermal annealing process with ambient oxygen, such as annealing at 600 °C for 30 min. It can also be formed through the deposition of a thin SiO_2_ layer. The generation of an oxide layer on the template surface increases the potential barrier height between Ag and template. It may decrease the migration rate of hot electrons from Ag NPs into template. However, its important function is to slow down the neutralization of Ag^+^ ions or to decrease the sticking probability, i.e., the probability of particle attachment when a diffusing particle collides with a cluster, in the DLA process. In this situation, the mean free path of hot electron in the template becomes larger for forming a more compact BT^19^. Fig. 14(a) shows the SEM image of an Ag nano-network structure, which consists of several major BTs. In fabricating this sample, before the formation of fresh Ag NPs, the GaN template is uniformly bombarded by Ar^+^ ions of 70 W in radio-frequency power for 10 sec to increase its point defect density. The fresh Ag NPs are formed by depositing Ag of 1.6 nm in thickness and thermal annealing at 140 °C for 30 min. The Ag NPs are then illuminated by the 395-nm LED for 16 hours under the condition of ~80% in ambient air humidity. Figure 14(b) shows a magnified SEM image of the Ag nano-network structure. Here, one can see a well-connected Ag pattern even though Ag coverage is low. The oscillating curve in Fig. 14(c) shows the transmission spectrum of this Ag network structure. From the smooth fitted curve, one can see that in the wavelength range of 400–2000 nm, the transmittance is always higher than 81%. Here, the depression of the red curve in the visible range indicates that an LSP resonance feature still exists in the network pattern. The high transmission level shown in Fig. 14(c) satisfies the application requirement of transparent conductor. However, the measured sheet resistance of this sample is as high as ~1300 Ω/sq. Although the surface Ag nano-network structure with such a high resistance level can still find application as a transparent heater, the reduction of resistance can help in finding a larger scope of application. Generally speaking, a denser fresh Ag NP structure, which can be formed by depositing a thicker Ag film, can lead to lower resistance but lower transparency. In one of our implementations, sheet resistance can be reduced to ~480 Ω/sq. Nevertheless, the transmittance of this sample decreases to a level between 70 and 75% in the visible range. Depending on application, a compromise between sheet resistance and transparency needs to be made.

## Discussions – Potential Applications

Currently, the most widely used transparent conductors are oxides, including indium-tin-oxide (ITO) and doped ZnO. However, they become absorptive in the UV-B and UV-C spectral ranges because of their bandgap limitations. They are also absorptive in the near-infrared (NIR) range due to their decreasing real parts and increasing imaginary parts of dielectric constants with increasing wavelength^28,29^. When the real parts of dielectric constants become negative, surface plasmon resonance leads to enhanced absorption in this spectral range. Therefore, a new material structure for transparent conductor applications in the deep-UV and NIR ranges is needed. The Ag nano-network structure demonstrated in this study has potential for this application. The transparency of such a surface metal nanostructure does not significantly change with wavelength, particularly in the UV and NIR ranges. As shown in Fig. 14(c), the transmittance in the NIR range is always higher than 83%. Because GaN is used as template in the current study, we cannot demonstrate the transparency in the deep-UV range. However, it is expected that the geometric-optics based transmittance in the UV range is no lower than that in the NIR range. The other advantageous feature of such an Ag nano-network structure for transparent conductor application is its low temperature fabrication. Although in some cases we use a thermal annealing process at a temperature between 100 and 200 °C for forming fresh Ag NPs, this process can be skipped because Ag NPs can be naturally formed without thermal annealing if Ag deposition is thin. Without the annealing process, the whole procedure for fabricating an Ag nano-network structure is undertaken at room temperature. The low-temperature process implies the potential of using this technique for fabricating a transparent conductor in a flexible device. In this application, the demonstrated Ag nano-network structure also has the advantage of tighter contact with template, when compared with the technique of laying synthesized Ag nanowires on top of a template^30–32^. As a transparent conductor, the required sheet resistance depends on application^33^. For the use as a transparent heater, sheet resistance over 1000 Ω/sq is acceptable. For touch panel application, sheet resistance needs to lie in the range of hundreds Ω/sq. In the application to a display device or solar cell, a sheet resistance level lower than 100 Ω/sq is required. Further improvements in the process of forming an Ag nano-network structure can result in a lower resistance level. The deposition of a thin layer of a conventional transparent conductor material, such as Ga-doped ZnO, at a low temperature can help in achieving a lower overall sheet resistance level^34^.

Although the DLA process has been widely studied theoretically and numerically, controlled experimental verifications are rarely reported, particularly for the two-dimensional DLA process. The formation of a surface Ag nano-network structure can provide us with a platform for the experimental study on the two-dimensional DLA process. A few parameters can be controlled for observing DLA behaviors, including the defect type and density of a centralized defect cluster, the density of distributed defects, the thickness of the surface oxide layer, the illuminating light wavelength and intensity, and the template material for controlling the potential barrier between metal and template and the electron mobility in the template. The variations of those parameters can lead to different DLA behaviors for verifying the theoretical models and simulation results.

## Conclusions

In summary, we have demonstrated the formation of a surface Ag nano-network structure on a GaN template, which consisted of multiple connected BTs formed through the DLA process. The DLA process was implemented through the settlement of diffuse Ag^+^ ions in a thin adsorbed water layer on the sample surface, which was regulated by hot electrons transporting in the template near its surface. The Ag^+^ ions were generated by UV light illumination onto the surface Ag NPs after the excited hot electrons overcame a potential barrier and migrated into the template. Either naturally formed threading dislocations or man-made defect clusters in the template could attract hot electrons and serve as the centers for forming BTs in the DLA process. The potential of applying such an Ag nano-network structure as a transparent conductor was discussed. However, further improvements in the fabrication procedure for reducing sheet resistance are required before practical applications can be implemented. Finally, it deserves mentioning that the process of forming an Ag nano-network structure is a useful platform for experimentally studying two-dimensional DLA behaviors.
